# Silica-bound benzoyl chloride mediated the solid-phase synthesis of 4*H*-3,1-benzoxazin-4-ones

**DOI:** 10.3762/bjoc.5.13

**Published:** 2009-04-23

**Authors:** Kurosh Rad-Moghadam, Somayeh Rouhi

**Affiliations:** 1 Chemistry Department, University of Guilan, P.O. Box 41335-19141, Rasht, Iran

**Keywords:** 4*H*-3,1-benzoxazin-4-one, silica-bound benzoyl chloride, solid-phase synthesis

## Abstract

The solid-phase synthesis of 4*H*-3,1-benzoxazin-4-ones was achieved using silica-bound benzoyl chloride (SBBC) as dehydrating agent in reaction with 2-acylaminobenzoic acids. The silica-grafted reagent (SBBC) was simply recovered after reaction and reused several times.

## Introduction

Solid-phase approaches are gaining more and more applications in organic synthesis mainly because reactions with a loaded substrate can be manually sequenced and driven to higher yields by using excess reagents, which are subsequently removed by simple filtration [1–7]. Conventionally, solid-phase synthesis referred to approaches in which the substrate was loaded onto the solid support and after programmed reactions the product was formed and then cleaved from the support. However, the concept of solid-phase chemistry is now used in a wider sense to cover alternative solid-phase reagent chemistry. In these approaches, substrates are chemically bound to the surface of the solid-supported reagent and converted to the product during liberation from the solid [8]. In another version, the core structure of the library molecule resides in solution and solid-phase reagents are added to the mixture to facilitate the reaction [9]. The solid-phase reagent or its residue is then removed from the heterogeneous reaction mixture by filtration. Besides functionalized organic polymers a variety of silica gel-grafted reagents have also been devised for solid-phase organic syntheses [10–14]. Compared with organic polymers, silica gel is more robust to drastic conditions and harsh reagents.

4*H*-3,1-Benzoxazin-4-ones are the most frequently used precursors in the synthesis of biologically active quinazolin-4(3*H*)-ones [15]. In addition, some of the 4*H*-3,1-benzoxazin-4-ones themselves have shown interesting biological activities [16–19]. As a result of these attractive features, a number of approaches have been developed for the synthesis of 4*H*-3,1-benzoxazin-4-ones. A literature survey regarding the synthesis of 2-substituted 4*H*-3,1-benzoxazin-4-one derivatives disclosed a variety of methods, including cyclodehydration of *N*-acylanthranilic acids by acetic anhydride [20], reaction of anthranilic acid with acid chlorides [21], treatment of methyl *N*-aroylanthranilates or methyl 2-ureidobenzoates with concentrated sulfuric acid [22], rearrangement of *o*-nitrophenylacetic acid in boiling acetic anhydride [23], condensation of 2-azidobenzoic acid with aldehydes [24], photoisomerization of 2-arylisatogen [25], and cyclocondensation of anthranilic acid with orthoesters [26]. Meanwhile, the cyclodehydration of *N*-acylanthranilic acids in refluxing acetic anhydride has been the prevalent method used for production of 4*H*-3,1-benzoxazin-4-ones. However, use of traditional dehydrating agents like acetic anhydride or sulfuric acid usually suffers from difficulties encountered in their handling, separation, and recovery after the reaction and may lead to environmental pollution. To eliminate these disadvantages we intended to evaluate the silica-bound benzoyl chloride (SBBC) as a solid-phase reagent for cyclodehydrating *N*-acylanthranilic acids **2a**–**f**. Accordingly, we introduce it here as an efficient and recyclable dehydrating agent for convenient synthesis of 4*H*-3,1-benzoxazin-4-ones.

## Results and Discussion

**Scheme 1:**
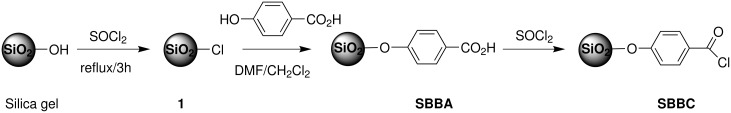
Three-step synthesis of silica-bound benzoyl chloride.

As is shown in Scheme 1, an approach consisting of three steps was employed for synthesis of SBBC through which first the surface of silica gel was converted to silica chloride by means of reaction with thionyl chloride [27–28]. The silica chloride was heated with 4-hydroxybenzoic acid to form the silica-bound benzoic acid (SBBA). The SBBA was washed with acetone to remove excess 4-hydroxybenzoic acid. Finally, the silica-bound benzoyl chloride, SBBC, was obtained from the reaction of SBBA with thionyl chloride. The IR spectrum of SBBA revealed a band around 1660 cm^−1^ clearly indicative of C=O stretching vibration of the carboxyl groups while the absorbtion of C=O stretching vibration of carbonyl chloride groups in SBBC appeared at 1704 cm^−1^.

The extent of functionalization was roughly determined gravimetrically by measuring the weight gain of SBBC relative to the starting silica chloride. This measurement gave an averaged value of 1.1 mmol of supported benzoyl chloride per gram of SBBC. There is a considerable difference between the measured amount of immobilized benzoyl chloride functions on the silica and the value of 2.3 mmol Si-Cl groups per gram of silica chloride as determined by Volhard’s precipitation titration [29] of Cl^−^ ions released on hydrolysis of 1 g silica chloride. Apparently, owing to porous nature of silica chloride, a significant fraction of the silyl chloride linkers remains unreacted even in treatment with excess 4-hydroxybenzoic acid during the loading process. On the other hand, and for the same reason, many of the graft sites may be unavailable or more hindered for reaction with the substrate molecules. In view of this we planned to determine the optimal amount of SBBC needed in reaction with 2-acylaminobenzoic acid experimentally, and to this purpose the reaction with 1 mmol of 2-benzoylaminobenzoic acid in refluxing dichloromethane was used as a model reaction. Preliminary experiments employing different amounts of SBBC in the model reaction showed that the best result in term of product yield is obtained from the optimum 2 g of SBBC. Thus, the reaction between 2-acylaminobenzoic acid (1 mmol) and 2 g of SBBC in refluxing dichloromethane went to completion in reasonable time and in fairly good yields (Table 1).

**Table 1:** Results of reaction between **2a**–**f** and SBBC.

Product	R	Reaction time (min)	Yields (%)^a^	Mp/°C	Lit. mp/°C

**5a**	CH_3_–	55	71	80–82	80–81^b^
**5b**	CH_3_CH_2_–	55	69	84–86	85–86^b^
**5c**	CH_3_CH_2_CH_2_–	55	72	58–60	59–60^b^
**5d**	Ph-	90	79	120–122	123–124^b^
**5e**	2-Furyl	90	78	99–100	102^c^
**5f**	Ph-CH=CH-	90	76	146–147	148^c^

^a^Yields of pure isolated products base on 2-acylaminobenzoic acid; ^b^[18]; ^c^[19].

The progress of reaction was monitored by TLC using ethyl acetate-petroleum ether as eluent. After completion of reaction the hot slurry of reaction-mixture was filtered to separate the SBBC residue. The residue was washed with dichloromethane and recovered by refluxing in thionyl chloride. Also, interesting to note is that under similar conditions and reaction times the treatment of 2-acylaminobenzoic acids with silica chloride **1** did not furnish the corresponding benzoxazinones.

To investigate the recoverability of SBBC its reaction with 2-benzoylaminobenzoic acid was chosen as the model. After completion of the model reaction the solid support was separated by filtration, washed with dichloromethane, dried in oven and then refluxed in excess thionyl chloride. The recovered SBBC was subjected to the next run of the model reaction. The results of the first experiment and subsequent experiments were almost consistent in yields after three runs (79%, 76%, 76%).

**Scheme 2:**
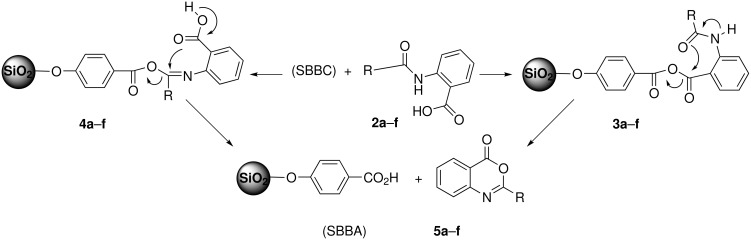
Plausible mechanisms for the synthesis of benzoxazinones **5a**–**f**.

However we have not determined the mechanistic details of reaction between SBBC and 2-acylaminobenzoic acids, plausible rationalizations may be advanced to explain the formation of benzoxazinones **5a**–**f** (Scheme 2). Presumably, these reactions involve the formation of the transient mixed anhydrides **3a**–**f** or **4a**–**f** which undergo thermal cyclo-elimination through intramolecular nucleophilic substitution to produce the benzoxazinone products leaving SBBA aside.

SBBC is a reactive reagent and is prone to hydrolysis but in comparison with commonly used dehydrating acid chlorides such as acetyl chloride and benzoyl chloride which are more hygroscopic and difficult to handle, it has negligeable vapour pressure so can be used simply. In addition, SBBC is not lost to environment as it easily is separated and recovered after reaction.

All the products are known compounds with IR, and ^1^H NMR spectral data in good agreement with their structures as well as with authentic samples prepared from the previously reported methods.

## Conclusion

A new solid-phase synthesis protocol has been developed for preparation of 2-substituted 4*H*-3,1-benzoxazin-4-ones. The main advantages of this process are the recyclability of the solid support and self release of products from the linker as a concomitant with their formation. Therefore, good yields of the products are obtained under relatively mild conditions by an economically viable and environmentally friendly method.

## Experimental

Melting points were measured on an electrothermal apparatus and are uncorrected. IR spectra were obtained in KBr wafers on a Shimadzu IR-470 spectrometer. Chemicals were obtained from Merck (Darmstadt, Germany) and were used without further purification. The required 2-acylaminobenzoic acid substrates **2a**–**f** were prepared according to the reported methods [18,30].

### Procedure for preparation of silica-bound benzoyl chloride (SBBC)

To 5.0 g of a well dried fine powder of silica gel 60 (0.063–0.200 mm) in a flask was added 25 mL of SOCl_2_. The mixture was refluxed for about 3 h, and then distilled to remove the excess SOCl_2_. To 4.0 g of the resulting white powder of silica chloride, a solution of 4-hydroxybenzoic acid (2.0 g) in CH_2_Cl_2_ (30 mL) and DMF (4 mL) was added and the mixture was refluxed for about 6 h. The suspension was filtered to separate the SBBA powders, the precipitate was washed with acetone (2 × 20 mL) and dried at 100 °C to give 4.43 g of SBBA. To 4.4 g of SBBA was added 25 mL of SOCl_2_. After refluxing for about 3 h, the excess SOCl_2_ was removed at reduced pressure and the resulting white powder of SBBC (4.64 g) was stored in a dry bottle.

### General procedure for preparation of 2-substituted 4*H*-3,1-benzoxazin-4-ones (**5a**–**f**)

To a mixture of 2-acylaminobenzoic acid (1 mmol in each case **2a**–**f**) in dry CH_2_Cl_2_ (12 mL) was added 2 g of SBBC (ca. 2.2 mmol reagent). The mixture was refluxed according to the time mentioned in Table 1, and then was filtered while hot. The filtered solids were washed with CH_2_Cl_2_ (2×10 mL). The combined CH_2_Cl_2_ fractions were evaporated and the residue was purified by recrystallization in n-heptane or by chromatography (in the case of **5f**) on silica gel using petroleum ether:ethyl acetate (2:1) as eluent to give the benzoxazinones (**5a**–**f**). The residue of SBBC was renewed by washing with 5 mL of dichloromethane, drying at 60 °C, and then refluxing (3 h) in 10 mL of SOCl_2_. The excess SOCl_2_ was removed under reduced pressure.
